# Abdominal Cutaneous Thermography and Perfusion Mapping after Caesarean Section: A Scoping Review

**DOI:** 10.3390/ijerph17228693

**Published:** 2020-11-23

**Authors:** Charmaine Childs, Hora Soltani

**Affiliations:** College of Health, Wellbeing and Life Sciences, Sheffield Hallam University, Sheffield S10 2BP, South Yorkshire, UK; h.soltani@shu.ac.uk

**Keywords:** infrared thermography, abdomen, cutaneous, temperature, childbirth, caesarean section, scoping review, perforator vessels

## Abstract

**Introduction:** Caesarean section (CS) is the most prevalent surgical procedure in women. The incidence of surgical site infection (SSI) after CS remains high but recent observations of CS wounds using infrared thermography has shown promise for the technique in SSI prognosis. Although thermography is recognised as a ‘surrogate’ of skin perfusion, little is known of the relationship between skin temperature and skin perfusion in the context of wound healing. **Aim:** To assess the extent of literature regarding the application of infrared thermography and mapping of abdominal cutaneous perfusion after CS. **Methods:** Wide eligibility criteria were used to capture all relevant studies of any design, published in English, and addressing thermal imaging or skin perfusion mapping of the abdominal wall. The CINAHL and MEDLINE databases were searched, with two independent reviewers screening the title and abstracts of all identified citations, followed by full-text screening of relevant studies. Data extraction from included studies was undertaken using a pre-specified data extraction chart. Data were tabulated and synthesised in narrative format. **Results**: From 83 citations identified, 18 studies were considered relevant. With three additional studies identified from the reference lists, 21 studies were screened via full text. None of the studies reported thermal imaging and cutaneous perfusion patterns of the anterior abdominal wall. However, two observational studies partially met the inclusion criteria. The first explored analysis methodologies to ‘interrogate’ the abdominal thermal map. A specific thermal signature (‘cold spots’) was identified as an early ‘flag’ for SSI risk. A second study, by the same authors, focusing on obesity (a known risk factor for SSI after CS) showed that a 1 °C lower abdominal skin temperature led to a 3-fold odds of SSI. **Conclusion:** There is a significant gap in knowledge on how to forewarn of wound complications after CS. By utilising the known association between skin temperature and blood flow, thermographic assessment of the wound and adjacent thermal territories has potential as a non-invasive, independent, imaging option with which to identify tissue ‘at risk’. By identifying skin ‘hot’ or ‘cold’ spots, commensurate with high or low blood flow regions, there is potential to shed light on the underlying mechanisms leading to infective and non-infective wound complications.

## 1. Background

Swift recovery with uncomplicated wound healing after caesarean section (CS) is important for women and their families to enhance the birth experience and to optimise care.

Of the estimated 234 million major surgical procedures undertaken worldwide every year [1], CS is the most frequent surgery in women [2], rising globally year on year [3,4]. Recent data from 169 countries (98.4% of births worldwide) estimate 29.7 million CS births annually, double the number (16 million) in 2000 [3,4]. In the EU (2018), CS birth reached 1.4 million [5]. One factor shown to contribute to higher CS rates is maternal obesity [6,7,8], increasing incrementally for both elective and emergency CS with each body mass index (BMI) category [9]. Compared to women of normal weight, obese pregnant women, particularly those with morbid obesity, are more likely to present with co-morbidity and suffer obstetric complications [9,10]. In the postpartum period, surgical wound complications such as surgical site infection (SSI) [11] and wound dehiscence are rising [12], with significant impact on health care costs [13,14].

Although infections after surgery can originate in organ spaces and deep tissue [15], most wound infections are superficial (96%), affecting skin and subcutaneous tissue only [16]. After CS, SSI rate varies depending upon methods of reporting and country of study [17,18,19]. Overall, the SSI rate in England is reported as 9.6% [11], with highest rates in obese women (BMI > 30 Kg/m^2^) ([20]; up to 50% in the morbidly obese [21]. Adiposity, therefore, appears to play a key role in susceptibility to wound infection. For example, Soper et al. [22] showed that it is abdominal subcutaneous tissue thickness (rather than weight or BMI per se) that represents a significant infection risk factor in the region of the abdominal wound incision after hysterectomy and CS. This suggests that the thicker the subcutaneous tissue, the greater the risk that infection may arise. By contrast, Vermillion et al. [23] showed that intraoperative measurement of subcutaneous tissue thickness was not a useful diagnostic test for later postoperative infection. There seems, therefore, to be uncertainty around the impact of subcutaneous tissue thickness as a ‘diagnostic’ for later wound infection. Nevertheless, the role that subcutaneous fat may play in the aetiology of postoperative wound complications after caesarean section merits further investigation. Here, the application of thermal imaging (as a surrogate for cutaneous perfusion) could be of benefit to clinicians in the context of CS, where decisions are made about subcutaneous closure, a surgical procedure shown to reduce the risk of wound disruption particularly in women with an incision depth of more than 2 cm [24].

In the absence of any direct measurement of subcutaneous fat thickness, BMI is frequently used to assess SSI risk after caesarean section. In a recent study of obese women giving birth by CS [25], long-wave infrared (LWIR) thermography mapping of anterior abdominal wall and wound site temperature suggests a potential independent imaging method to stratify women to risk of SSI based on skin and wound temperature.

Infrared thermography, used in the biomedical field since the 1960s [26], has the advantage of being a non-invasive, non-ionising, non-contact imaging solution to the measurement of skin temperature across the body surface. It provides an image of the distribution of surface temperature in the form of a heat map. Numerous pathophysiological processes lead to changes in the distribution of heat at the body surface such that skin temperature rises or falls principally due to fluctuations in cutaneous blood flow. Under controlled conditions, skin temperature assessed by thermography has been used in plastic surgery and shown to perform well as a surrogate for the assessment of cutaneous perfusion through the identification of arterial perforator vessels, with their location visible on thermography as skin ‘hot spots’ [27].

### 1.1. Context

Mapping of cutaneous perfusion is a fundamental requirement for successful pre-operative surgical planning of free flaps. In reconstructive surgery, thermal imaging has proven to be a relatively simple and inexpensive imaging solution to select the most suitable perforator vessel to perfuse the flap. In the context of routine postoperative wound care, however, perfusion of the wound and adjacent body regions is not easily assessed at the bedside with standard radiological techniques. Nevertheless, perfusion assessment for early detection of poor cutaneous blood flow could alert clinicians to microvascular changes as a marker of tissue viability [28]. In the context of this scoping review, the focus is to identify the breadth of studies pertaining to incisional wounds, specifically relating to the correspondence between abdominal wall cutaneous temperature using infrared thermography and abdominal cutaneous perforator vessel blood flow in an obstetric population—women undergoing CS birth.

### 1.2. Concept

The transverse Pfannenstiel (supra pubic) incision is one of the most popular surgical incisions for performing CS and is cosmetically acceptable in modern obstetrics [10] across all BMI categories, including those women with a large abdominal panniculus [29]. Blood supply to the abdomen in the region of the Pfannenstiel incision originates from the superficial inferior epigastric artery, superficial external pudendal artery, and, most caudal, perforators of the deep inferior epigastric artery DIEA) [30,31]. The superficial external pudendal artery enters tissue in the region below the incision. The flow of blood in these vessels supplies the subdermal plexus and skin and is the principal route determining skin temperature around the CS incision.

Clinicians recognise that healing is delayed if blood flow to the wound is poor, not least with respect to the supply of oxygen to the wound bed [32,33,34]. By exploring abdominal wall cutaneous temperature using thermal imaging, the goal is to shed light on subtle changes in cutaneous perfusion to the wound (and adjacent tissue) at early times after surgery to forewarn the clinician of later wound complications (infective and non-infective origin). The concept underpinning this review, therefore, is that knowledge of the relationship between emitted long-wave thermal radiation, skin temperature [35] and blood flow in the region of the abdominal incision may offer a practical bedside imaging solution to broaden wound assessment criteria from the visible spectrum into the infrared region. Looking at the wound map in ‘infrared’ and measuring the temperature of abdominal territories [36] offers a new perspective on wound assessment with an easily performed technique as a ‘surrogate’ approach to explore the cutaneous microcirculation.

### 1.3. Aims and Objectives

In view of international concerns for the rise in CS [37] and the prevalence of SSI [38] amid knowledge of the impact of skin blood perfusion on wound healing [39], the overall aim of this scoping review was to examine the literature on the application of infrared thermography mapping of abdominal cutaneous perfusion after birth, particularly among women following CS across the range of maternal body mass index (BMI).

## 2. Methods

The methodological framework of Arksey and O’Malley [40] was adopted to map key concepts and the underpinning research outcomes across the breadth of published literature. Specifically, this review seeks to address one of the reasons suggested by the authors [40] for undertaking a scoping review: to identify gaps in the literature where there is limited research on the topic of interest.

### 2.1. Search Strategy

To report this scoping review, the “Preferred Reporting Items for Systematic Reviews and Meta-Analyses extension for Scoping Reviews (PRISMA-ScR)” [41] was followed. Two major databases including CINAHL and MEDLINE were searched. Search terms and synonyms were merged using Boolean operators under four facets “maternal OR postpartum”, “abdominal skin”, “blood flow or perforators” and “thermal imaging” (Appendix A). An additional search of reference lists of key articles was carried out plus contacting experts in the field for further relevant literature. No date restrictions were used, with databases searched from inception to 13th May 2020. Studies were limited to those conducted in humans, female and published in the English language. The search scope was purposefully kept inclusive of all types of research design related to childbirth to ensure capture of all relevant publications.

### 2.2. Eligibility Criteria

Due to the exploratory nature of the review, the objective was to ensure capture of a wide scope of relevant literature. Articles were considered for inclusion if:

1. The participants were postpartum women,
2. Infrared thermal imaging of the abdomen was undertaken,
3. Abdominal blood flow mapping was reported, and
4. The article was published in the English language.

Studies on animals or male participants were excluded.

Selection of Sources of Evidence: To ensure consistency, two authors screened all publications, discussed the results and amended the screening and data extraction manual before beginning screening for this review. Both reviewers working sequentially screened all citations for inclusion by title and abstract. Where citations were considered of potential relevance, the full text was read. To reach consensus on article content for inclusion, the reviewers discussed each article to reach mutual agreement.

### 2.3. Data-Charting Process

A draft data-charting form incorporating terms recommended by the Joanna Briggs Institute [42] provided the first iteration of the process towards a logical and descriptive summary of all full-text articles screened of the eligible studies aligned to the scoping review aim. A second, refining, step allowed the determination of specific details about the population, the medical discipline, study methods and key findings relevant to the aim of the scoping review. Reviewers independently extracted and charted the data, discussed the results and continuously updated the charting table (Appendix B) as an iterative process to produce a summary table of included (Table 1) and excluded (Table 2) studies.

## 3. Results

**Figure 1:**
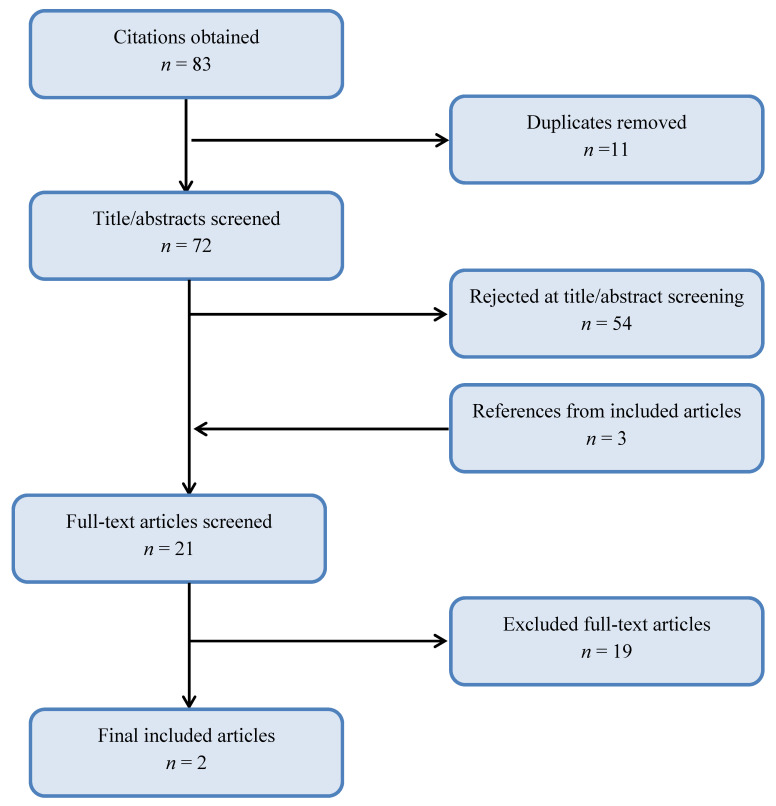
Flowchart of study selection (11 May 2020).

Figure 1 illustrates that our search yielded 83 citations. Upon removal of duplicates, a total of 72 titles and abstracts were screened individually by each reviewer with 54 rejected as not relevant to the scoping review. From this, 18 full-text articles along with an additional 3 articles identified from the bibliography, made a total of 21 articles screened. Upon reading, and data extraction of all the articles’ texts, not one fully addressed our review aim established at the outset of the scoping review. Table 1 presents characteristics of the included studies and Table 2 provides justification for the excluded studies, highlighting the current gap in the literature. None of the studies addressed the key elements of the inclusion criteria or the link between infrared thermography of abdominal skin temperature and correspondence with cutaneous blood flow. However, two observational studies [25,43] addressed three of four key eligibility criteria specifically related to infrared thermography of the abdomen in postpartum women but did not address cutaneous blood flow measurements concurrently (Table 1).

Description of included studies: Childs et al. [43] employed thermal imaging, in static mode, to develop and refine qualitative mapping and quantitative analysis techniques to define thermal territories of the human abdomen after surgery. Caesarean section was selected as a ‘model’ of incisional wounds. A convenience sample of 20 women after CS birth, aged 20–39 (median 33) years were recruited across all BMI categories; normal weight (BMI 18.5–24.9 kg·m^−2^), overweight 25.0–29.9 kg·m^−2^, and obese (≥30 kg·m^−2^). All but two women were admitted for elective CS. Long-wave infrared (LWIR) thermography was used to undertake quantitative temperature analysis of the territories of the abdominal wall in areas around and along the surgical incision using a FLIR^TM^ (FLIR systems, Täby Sweden) uncooled camera (T450sc) with pixel resolution, 320 × 240 (NETD < 30 mK). Camera calibration was undertaken against a certified blackbody source.

The first thermal images were undertaken 30 min after removal of dressings and before the women were discharged from hospital, typically 24–48 h postoperative. For image processing and analysis, different methods were explored to enhance the image and for edge detection of the greyscale thermogram for both wound and abdominal regions of interest (ROIs) and for temperature profiling along and around the wound. Hierarchical cluster segmentation (HCS) was used to analyse the heat map and to identify discrete abdominal skin and wound regions displaying as ‘hot’ or ‘cold’ spots on the thermal map. In this study, all methodological approaches were taken to ensure measurement accuracy with respect to camera focus, distance from camera to target, after camera calibration. Camera proprietary tools were used for quantitative analysis of temperature values, reported as mean values of wound site, mean of reference site (healthy abdominal skin) and temperature difference (reference minus wound). Hierarchical clustering-based segmentation of boundary regions were performed using techniques of Selvan [62]. Subtle variations in temperature clusters at wound and reference sites were observed. The HCS isotherm boundaries revealed the significant variability in thermal clusters that can be distinguished across the wound, clusters not visible on the greyscale maps. Imaging was performed at postnatal ward ambient conditions of 21–26 °C (median 24 °C). All women at the time of imaging were apyrexial.

Furthermore, by exploring analysis methodologies to ‘interrogate’ the thermal map, this study revealed ‘signature’ features of the incisional wound as a potential clinical application for early prediction of SSI. By thermal profiling, subtle differences across wound and adjacent sites, otherwise undetectable in routine postoperative care became apparent. The larger the difference in temperature between wound and reference site the greater the likelihood of later SSI. This study provides the first evidence of a surgical wound infection prodrome linked to a reduction in skin temperature in and around the wound. On qualitative review of the thermal map, the main observation was of ‘cold spots’, evident along the wound as early as day 2 postoperative. Whilst recognising the limitations of the small study sample, the authors suggest that the early appearance of temperature ‘cold spots’ along the wound, commensurate with low thermal radiation intensity suggests areas of avascular tissue and a probable early ‘flag’ for SSI. From narratives obtained from the women studied, the authors found thermal imaging to be a feasible technology at the bedside and one supported by the women themselves who reported that being able to ‘see’ images of the wound provided greater consistency than traditional wound assessment.

Further exploratory work [25] in a second cohort of (obese) women undergoing caesarean section, further characterised the temporal profile of the abdominal thermogram, this time during hospital admission (day 2) and throughout the first month after surgery. LWIR thermography of the wound and adjacent abdominal skin was made, in static mode, in the target population of 50 women aged 21–44 years (median 32 years) with BMI in the range of 30.1–43.9 kg·m^−2^ (median 34.2 kg·m^−2^) using a portable thermal camera, T450sc (FLIR, Täby, Sweden). Thermal imaging (pixel resolution 320 × 240) was performed during the woman’s in-patient stay and at each of three home visits up to day 30. The camera was calibrated against a blackbody source (P80P), Amtek-Land, Dronfield, UK) at temperatures between 30 and 45 °C and compared to measurements from a certified (UKAS, UK) independent thermometer, type 100 Ω platinum resistance thermometer (PRT 100, ISOTECH, Skelmersdale, UK.

In this series, 14 of 50 women (28%) had a confirmed GP diagnosis of SSI, typically made during the third week after surgery (median 18 days (range 6–24 days)). Results showed that visual assessment of the wound was poor with lack of agreement between clinicians in their subjective assessment of the wound plus a lack of agreement in identifying wounds most likely to become infected. By contrast, quantitative thermal imaging of the wound and abdominal temperature improved predictions for later wound infection. Best-fit of the statistical model was at day 7 postoperative: the wider the temperature difference between wound and abdomen the greater the odds of infection. Overall, wound temperature was consistently higher than at the abdomen. Women who later developed SSI had the lowest mean abdominal temperature, making a substantive contribution (at day 2) and day 7 to an increased risk of infection in the obese cohort of women. A 1 °C lower abdominal temperature in those who later developed SSI had 3-fold increased odds of wound infection. These temperature data fit, on qualitative analysis of the thermal map, with the convergence of multiple cold spots forming a ‘cold’ abdominal ‘thermal topography’, a signature which emerged as an independent risk factor for SSI before any ‘overt’ signs of SSI were apparent.

## 4. Discussion

This scoping review, through a systematic and extensive search of literature, has demonstrated a gap in the literature on the use of infrared thermal mapping of abdominal skin perforator vessels to identify areas of high (or low) blood perfusion in an obstetric population. However, this review did identify two recent studies which sought to interrogate postoperative abdominal thermal territories in the region of Pfannenstiel incision. Here, we may speculate on the role of thermography as a potential surrogate for cutaneous perfusion in and around the surgical wound.

It is now over 50 years since Kliot and Birnbaum [59] sought to describe the infrared profile of wound healing after Pfannenstiel incision in women undergoing gynecological surgery. Even allowing for the infrared camera technology resolution and accuracy of the day, imaging revealed a thin ‘cold line’ across the incision, which, in uncomplicated wounds, began to ‘blend’ with the wound heat pattern such that the cold line was lost by days three to four after surgery. Although Kliot and Birnbaum [59] did not mention the temporal changes observed in ‘complicated’ wounds, our group have previously demonstrated the link between a series of incision line ‘cold spots’ observed early after surgery and later SSI in two separate study cohorts—closure of enterostoma [36,63] and after CS [25,43]. Anterior abdominal wall skin temperature is significantly lower during the first seven days after surgery in obese women who later develop SSI compared to those whose wounds healed without complication [25]. Lower abdominal skin temperature together with ‘cold spots’ along the incision may reflect microvascular changes in the obese person leading to a reduction in skin perfusion because (i) low skin temperature across the abdominal wall is consequent upon increased insulating capacity of subcutaneous fat and (ii) ‘cold spots’ along the incision may represent changes in perfusion dynamics of the wound representing poor wound vascular perfusion. In other words, the lower the cutaneous (and wound) temperature the greater the risk of poor tissue perfusion and subsequent wound infection.

It is now known that the impact of adiposity has the effect of lowering skin temperature. Although not studied in an obstetric population, studies by Chudeka et al. [48] and Savastano et al. [45] do provide insight of the impact of body composition on skin temperature. For example, Chudeka [48] focused on the implications of obesity on skin surface temperature distribution mapped across the abdomen using infrared thermography. Abdominal skin temperature was shown to be lower in obese, compared to normal weight, adults. In women specifically, both Chudecka et al. [48] and Savastano et al. [45] report that an increase in subcutaneous abdominal fat leads to an increase in insulation and a reduction in heat transfer from body core to skin, so producing lower abdominal temperature values. Whilst no difference in core temperature was reported in the study groups (normal weight vs. obesity) the decreased heat dissipation via abdominal skin in the obese group was offset by an increase in skin temperature at the extremities (hands), so helping to maintain normothermia [45]. In obese women, high subcutaneous fat content creates an insulating barrier to heat exchange at the skin surface and this is particularly so in abdomen and thighs. The authors further suggest that variability in skin temperature due to adiposity is likely linked to location of cutaneous perforator vessels [64] which can be detected as ‘hot spots’ using infrared thermography [65]. Whilst cutaneous blood flow was not measured directly in these studies, the authors suggest that the relationship between abdominal adiposity and a concomitant increase in body insulation may be linked to differences in number and distribution of cutaneous perforator vessels in obese versus normal weight adults and may, therefore, explain a vascular component to differences in skin surface temperature observed between the two groups. As fat tissue mass expands without a corresponding increase in blood flow per cell, a relative hypoperfusion and decrease in tissue oxygenation [66] may occur increasing the risk for the development of avascular, non-viable tissue.

Whilst Jo and Kim [54] in their retrospective study also focused on skin temperature differences across the body of women in an obstetric population, the objective was to use infrared thermography to diagnose infertility based on the distribution of skin temperature in four regions of interest (ROIs); two each over the abdomen and axilla. The results showed that infertility was associated with significantly lower abdominal temperature. Several explanations were offered including an effect of perforator vessel blood supply in the region of the abdomen. Perforator vessels connect the vascular system of the abdominal muscles to subcutaneous tissue, terminating in the subdermal layer of the skin [67]. As it is known that skin temperature is primarily dependent upon blood supply [68], low skin temperature of the abdomen suggested that infertility is associated with poor vascular perfusion to the skin with infrared thermography a potentially valuable infertility screening modality [54]. Although cutaneous perfusion was not measured directly, this study contributes to the scoping review aim by further endorsing the contribution of infrared thermography as a surrogate for cutaneous arterial perfusion.

In studies by Childs et al. [43], mapping of abdominal thermal territories in postpartum women undergoing CS revealed a similar signature of wound infection previously observed by Siah and Childs [36] in major abdominal surgery. After CS, blood supply to abdominal skin in the region of the Pfannenstiel incision is supplied from multiple cutaneous perforators arising from underlying source vessels that pierce fascia to supply the skin [69]. These perforator vessels can be identified in a number of ways including, magnetic resonance imaging [70], CT angiography [71] indocyanine green fluoresecent angiography [71], colour Doppler imaging [69] but all require significant resources and/or training. None are practical for routine monitoring at the bedside. LWIR thermography is an alternative imaging technique and, being a non-ionising and relatively inexpensive imaging modality has also been used for locating dominant perforator vessels in flap surgery, in conjunction with Doppler [72,73].

Under ‘normal’ ambient conditions, skin temperature is a thermal mosaic [43,56]. In ‘static’ mode, the thermal image represents the sum of all factors (including environmental temperature artefacts) which can have an impact on the observed skin temperature pattern [73]. To overcome this, de Weerd et al. [27,72] introduced the concept of dynamic infrared thermography (DIRT) in pre-operative planning of deep inferior epigastric perforator (DIEP) flaps. Here, cooling the skin surface with a fan (or application of a cold object to the skin) produces visible changes in the thermal image such that temperature variability is diminished to a near equivalent temperature. This method of recovery-enhanced thermography, first examined by Itoh and [74] Arai [65] is now an accepted method to localise cutaneous perforators [75]. On removal of cold challenge and upon a short (5 min) period of recovery, presence of the perforator vessel corresponds with first appearing hot spots [72] with a ‘warm halo’ indicating the immediate area of perfusion [73]. The associated arterial Doppler sound confirms the selection of the vessel as a suitable perforator for free-flap harvesting on ‘gold-standard’ multidetector row computed tomography (MDCT) [76]. In a recent systematic review [77] the authors conclude that DIRT whilst still relatively novel in the surgical sphere will attract interest in the years to come particularly as the technique is easy to use and less complex than standard radiological imaging for locating skin perforators.

Although the focus of attention and application of DIRT is in locating perforator vessels of the anterior abdominal wall in the planning of autologous free-flap harvesting for breast reconstruction [27,78,79], this principle, applied to surgical wounds, could provide surgeons in other specialties with a greater understanding of the likelihood of later wound complications consequent upon disruption to the vascular supply, a potential marker for impaired tissue perfusion and slow healing in the region of the wound.

Our aim in undertaking this scoping review was to gauge the breadth of literature available to demonstrate evidence of a relationship between thermal imaging of abdominal wall temperature and cutaneous perfusion in the setting of postoperative wound healing after CS. During this early period, wound inflammation and infection are the primary concerns of the clinician in the context of wound healing and tissue viability. During inflammation, it is recognized that local skin blood flow increases and with it a concomitant increase in temperature [80,81] By contrast, the significance of skin surface ‘cold spots’ on thermography appears to have attracted much less attention for its prognostic potential.

Of the limited number of studies available using infrared thermography in obstetric populations, almost all have been confined to static imaging. By contrast, Falzon et al. [44] were the first to use dynamic thermography (in pregnant and non-pregnant women) to improve the imaging technique; the aim being to yield trends and spatiotemporal image characteristics that may be missed on passive (static) imaging. The existing literature shows that static infrared thermography is the technique used in the majority of studies identified. When used after CS, thermography of the wound and neighbouring region and the heat signature from the abdominal map appears to show some promise as to the possible vascular mechanisms involved in early wound infection particularly in the group at highest risk of SSI: obese women.

## 5. Conclusions

This scoping review confirmed a gap in knowledge regarding correspondence between postoperative abdominal wall skin thermal mapping and skin perfusion after CS. There is a need, therefore, for further research in this field to establish the potential clinical relevance, during routine surgery, of knowing the relationships between abdominal skin temperature ‘hot’ as well as ‘cold’ spots. Correspondence with changes in cutaneous perfusion via independent methods of blood flow measurement (e.g., Doppler) could enhance our understanding of the significance of the thermal signature in the acute surgical wound. Extending assessment of the surgical wound from the visible to the infrared spectrum may have value in identifying the vascular impact of surgery in the region of the wound on local cutaneous perfusion and subsequent wound healing and infective complications.

## Figures and Tables

**Table 1:** Included studies.

Author	Title	Design, Setting, Methods	Sample Size/Participants	Findings
Childs et al., 2016 [43]	Thermal territories of the abdomen after caesarean section birth: infrared thermography and analysis	Design: prospective feasibility and exploratory study Setting: postnatal wards, thermography on day 2 postoperative Methods: Body Temperature: tympanic membrane Ambient Conditions: air temperature, relative humidity, air velocity (postnatal ward). Thermal Imaging Equipment: LWIR using FLIR™ 450sc uncooled camera in static mode. Pixel resolution 320 × 240, NETD <30 mK, accuracy ±2 °C, spatial resolution 1.36 mRAD (milliradians) Calibration: Certification via blackbody source (FLIR™ Systems) Statistical analyses: SPSS V22 (IBM) Qualitative: Patient narratives from participants on perspectives of acceptability of thermal imaging at the bedside	Convenience sample 20 women by BMI group Women undergoing elective CS birth All BMI categories; normal weight: 18.5–24.9 kg/m^2^, overweight: 25.0–29.9 kg/m^2^, obese: >30 kg/m^2^	Recruitment: women aged 20–39 (median 33) years; obese n = 8, overweight n-7, normal weight n = 5. All participants apyrexial at time of imaging. Temperature (°C) profiling: of abdomen (healthy reference site and wound). HCS pixel clustering of the incision and healthy adjacent region revealed differences in the thermal territory profiles (scar and adjacent region) Maximum temperature difference between healthy reference abdominal skin and wound site exceeds 2 °C in those who were suspected of developing SSI. For the majority (n = 16) small differences in wound site and reference site temperature were observed Outcome: this study shows that temperature differences between wound and reference skin territories could provide a thermal ‘signature’ of possible infection risk with ‘cold spots’ proposed as a putative marker of an ‘avascular’ region along the incision site Women reported positive feedback. LWIR is an acceptable technology as a future wound surveillance technology. The study meets some but not all inclusion criteria, e.g., postpartum women (with incisional wounds following caesarean section) with abdominal thermal mapping but does not include any measures of cutaneous blood flow in relation to abdominal temperature
Childs et al., 2019 [25]	The surgical wound in infrared: thermographic profiles and early stage test accuracy to predict surgical site infection during the first 30 days after caesarean section	Design: Prospective early stage test-accuracy study Setting: Postnatal wards and 30 day community follow-up Methods: Body Temperature: tympanic membrane Ambient conditions: air temperature, relative humidity, air velocity of postnatal ward and patient’s own room at home Wound Assessment: Centre for Disease Control (CDC) SSI criteria Antibiotic Prophylaxis: type/dose/duration documented (in patient) General Practitioner (GP) outpatient prescribing: type/dose/duration Thermal Imaging Equipment: LWIR using FLIR™ 450sc uncooled camera in static mode. Pixel resolution 320 × 240, NETD < 30 mK, accuracy ±2 °C, spatial resolution 1.36 mRAD (milliradians) Calibration: across temperature range 30–45 °C against blackbody source (P80P Amtek Land Instruments, Dronfield UK) Measurement Reliability: LWIR temperature measurements compared against independent certified (UKAS, UK) platinum resistance thermometer (type 100 Ω, platinum resistance thermometer (PRT) 100 Isotech, Skelmersdale UK) Imaging Protocol: LWIR thermography performed before hospital discharge (24–48 h postoperative) and on day 7, 15 and 30 after surgery Wound Visual Assessment: digital photographs taken alongside LWIR thermal image Follow Up: confirmed at 30 days postoperative via GP surgery	Obese women (BMI ≥ 30 kg/m^2^ at booking in) giving birth by elective or emergency CS Sample size: With an estimated SSI incidence (obese population) of 20%, test-accuracy for early stage identification of SSI to correctly identify women with (sensitivity) and without (specificity) infection, sample size: n = 50 women Abdominal skin regions of interest (ROIs) selected for each woman: ROI 1: Abdomen (umbilicus centrally) ROI 2: Wound site	Recruitment: 53 afebrile women aged 21–44 (median 32) years with BMI 30.1–43.9 median 34.2 kg/m^2^) recruited. Fifty women entered the study. First thermal image taken in hospital between day 1–3 (median 2) days postoperative. Full sets of thermal images taken on 4 occasions (one in hospital and three at home) were achived in 78% of sample Visual assessment: of the wound from digital photographs showed poor agreement with the eventual clinical wound outcome, indicating no improvement of wound assessment over chance. Agreement for the rater’s wound assessment for likely SSI indicated no consensus of opinion by clinicians At the 30 day postoperative follow-up time point, 16 women (32%) returned to the GP (6–24: median 10) days after surgery with suspected wound infection; one woman returning on two occasions. Fourteen women in this obese sample were given a clinical diagnosis of SSI (28%) Cutaneous temperature measurement: ROI 1 (abdomen) and ROI 2 (wound). Wound site (ROI 2) was consistently at a higher temperature than ROI 1 (abdomen). ROI 1 was at a significantly lower temperature in women who subsequently developed SSI. A unit (1 °C) reduction in abdominal temperature led to a 3-fold raised odds of infection. A 1 °C widening of temperature between ROI 2-ROI 1 (wound minus abdomen) was associated with an odds ratio for SSI of 2.25 (at day 2) and 2.5 (at day 7) Correct prediction for wound outcome using logistic regression models ranged from 70–79%. Thermal mapping: Although the wound was observed on the thermal image as ‘hotter’ than the abdomen, ‘cold spots’ were observed along the scar from day 2 in many women who later developed SSI. The origin of wound’ cold spots’ was proposed as attributed to a reduction in cutaneous blood flow. No direct measures of cutaneous blood flow were taken This study meets some but not all inclusion criteria. Infrared thermography was performed in obese women at high risk of postoperative wound infection (SSI). No confirmatory evidence was obtained to validate the link between low cutaneous temperature and tissue perfusion

**Table 2:** Justification for exclusion of retrieved articles.

Author	Title	Include	Reason
Falzon et al., 2018 [44]	Principal component analysis of dynamic thermography data from pregnant and non-pregnant women	NO	Although studies were undertaken in women (pregnant and non-pregnant) using infrared thermography of the abdomen as the region of interest (ROI) the study did not include measures of related cutaneous perfusion.
Savastano et al., 2009 [45]	Adiposity and human regional temperature	NO	Does not address all aspects of the inclusion criteria but does include infrared thermography mapping of abdomen but in the context of adiposity and body composition providing some background relevance to the research aim.
Chandra et al., 2004 [46]	A preliminary study of cutaneous blood flow associated with postpartum use of oral misoprostol	NO	Study aim was a randomized controlled trial to compare effects of oral misoprostol (a uterotonic drug with shivering side effects) with intravenous oxytocin in women at risk of postpartum haemorrhage. No abdominal thermography was performed but Doppler flux was used to assess changes in tissue perfusion (of triceps) postpartum temperature.
Ciantar et al., 2018 [47]	Registration of dynamic thermography data of the abdomen of pregnant and non-pregnant women	NO	This methodological paper, in an obstetric population, set out to find reliable methods to obtain accurate thermography images using dynamic mode whereas typically, this is undertaken in ‘static’ thermography mode. Changes in the ROI can be affected by movement over time, but dynamic mode eliminates sources of error in spatial alignment. Pregnant and non-pregnant (but not postpartum) women were included.
Chudecka et al., 2014 [48]	Body surface temperature distribution in relation to body composition in obese women	NO	This study used infrared thermography to compare profiles (total body and regional) between obese and normal weight women to identify body regions within which heat transfer is impeded. This work adds further supportive evidence that mean body surface temperature of the abdomen decreases as the percentage of body fat rises. No measures of cutaneous blood flow linked to abdominal infrared thermography.
Chudecka and Lubkowska 2016 [49]	Thermal imaging of body surface temperature distribution in women with anorexia nervosa	NO	The aim of this study was to assess the relationship between subcutaneous (and visceral) fat and skin temperature in 20 young women with Anorexia Nervosa (AN) compared to a reference group of healthy women. Infrared thermography was used to measure skin temperature in 12 body regions. Mean abdominal temperature in patients with AN was higher than the reference group. Higher skin (and abdominal) temperatures were attributed to significantly reduced subcutaneous fat content. No measurement or indication of cutaneous blood flow was undertaken in association with infrared thermography.
King et al., 2017 [50]	Thermography examination of abdominal area skin temperatures in individuals with and without focal epilepsy	NO	This study revisited an osteopathic theory that the abdomen of people with epilepsy manifest ‘cold spots’ due to inflammatory lesions originating with injuries to viscera and or the musculoskeletal system. Using infrared thermography to map abdominal regions, adults with focal-onset epilepsy had colder abdominal areas than controls. No measures reported of cutaneous blood flow in target abdominal regions.
McFarlin et al., 2015 [51]	Comparison of techniques for the measurement of skin temperature during exercise in a hot humid environment	NO	Thermal imaging was used as one of three methods for skin (abdomen and biceps) temperature measurement using wired skin electrodes and iButtons mounted on skin using adhesive tape, thermal imaging for temperature values. Limits of agreement between measures were performed. Differences between skin electrode, iButton and thermography were comparable for biceps but not abdomen. Underlying subcutaneous tissue was proposed as an explanation for large measurement bias with thermography skin temperature measurement. Not postpartum women.
Dutta et al., 2020 [52]	Lower extremity blood flow velocity in obese versus non-obese pregnant women	NO	The objective was to assess risk factors for venous thrombo-embolism using Duplex ultrasound in pregnant women during the third trimester, with and without obesity. No thermal imaging was undertaken and the method of blood flow estimation was lower limbs not abdomen. Blood flow in lower extremities was higher in obese compared with non-obese women.
Goodlin and Brooks 1987 [53]	Abdominal wall hot spots in pregnant women	NO	In a group of 140 women (non-pregnant and pregnant) an infrared temperature probe was used to ‘map’ seven regions of the abdominal wall. Hot spots were defined as temperatures ≥0.3 °C than the rest of abdomen. Left and right inguinal hot spots were most common (72%) followed by inguinal areas (58%) in normal pregnancy. Authors noted that whilst obese, non-pregnant, women had inguinal hot spots this was thought to be due to heat trapping by the abdominal pannus contributing to heat retention in this region. No postpartum or abdominal skin blood flow mapping was undertaken.
Jo and Kim 2016 [54]	Comparison of abdominal skin temperature between fertile and infertile women by infrared thermography: A diagnostic approach	NO	Abdominal skin thermographic profiles of fertile and non-fertile women (using an infrared camera) were compared, showing a higher temperature in fertile women. The authors comment that temperature difference between the groups could be due to blood perfusion differences, but no abdominal skin blood flow mapping was undertaken. The study population did not include postnatal women.
Hu et al., 2017 [55]	Combining laser Doppler flowmetry measurements with spectral analysis to study different microcirculatory effects in human pre diabetic and diabetic subjects	NO	Laser Doppler flowmetry (LDF) and spectral analysis was undertaken in adult males and females to establish microcirculatory regulatory mechanisms at the ankle skin in diabetic, pre-diabetic and ‘normal’ subjects. Results showed that endothelial, neurogenic and myogenic activities of blood vessels were all smaller in pre-diabetic or diabetic patients compared to ‘normal’. No measures of thermography nor cutaneous blood flow were performed at the abdomen and no postpartum women were studied.
Bruins et al. 2018 [56]	Thermographic skin temperature measurement compared with cold sensation in predicting efficacy and distribution of epidural anaesthesia	NO	Thermography was used as an alternative to ‘gold-standard’ cold sensation test of epidural anaesthesia. Thermographic imaging was performed in patients undergoing abdominal, thoracic and orthopoedic surgery. The decrease in skin temperature due to body heat distribution and vasodilation induced by epidural block was detected using thermography indicating that thermal imaging could provide an additional and objective assessment method to the cold-sensation test. No cutaneous blood flow measurements.
Siah and Childs 2016 [36]	Thermographic mapping of the abdomen in healthy subjects and patients after enterostoma	NO	Male and female adults were recruited in this study. Thermal patterns in nine abdominal regions and at the surgical wound (enterostoma) revealed some differences between healing and infected wounds. Healing wounds showed a trend in the thermal ‘map’; an increase in temperature on the first postoperative day, and ‘warming’ over the subsequent five days. ‘Cold spots’ emerged on the thermogram of the surgical wounds which subsequently became infected. Subjects with higher BMI had significantly lower mean abdominal temperatures in 4 of 9 regions suggesting that body composition influences skin temperature. Infected surgical wounds appear ‘colder’ than healing wounds. Postpartum women were not included in this study. No cutaneous blood flow mapping was undertaken by independent method such as Doppler.
Key 2014 [57]	Preliminary demonstration using localized skin temperature elevation as observed with thermal imaging as an indicator of fat-specific absorption during focused-field radiofrequency therapy	NO	This study investigated the safe duration of focused-field radiofrequency therapy skin heating to temperatures in excess of 40 °C. Five subjects only studied. Sample did not include pregnant or postpartum women. Peak heating of skin (hottest spots) were abdomen and flank where areas of subcutaneous fat were greatest and where visible fat was ‘pinchable’.
Willman 1973 [58]	Pitfalls of Abdominal Thermography	NO	A qualitative review of body heat patterns using a ‘thermograph’ system. Images were produced on polaroid film. Four inflammatory conditions in the abdominal region were investigated to determine similarities of the thermograph. Temperature change and underlying disease was observed, particularly of the upper right quadrant. Participants were male and female but none were postpartum and no measures of cutaneous blood flow were performed at sites of interest.
Kliot and Birnbaum 1964 [59]	Thermographic studies of wound healing	NO	Pre- and postoperative abdominal thermography was used in different populations of patients undergoing gynaecological, pelvic and abdominal surgery to follow phases of uncomplicated wound healing. The study provides insights of classical wound healing stages in relation to what is seen on thermography, i.e., scar looks ‘cold’ along the incision. The scar ‘cold’ spot gradually disappears, ‘blending in’ to surrounding abdominal skin. No direct measures of cutaneous blood flow.
Gershon-Cohen et al., 1965 [60]	Obstetric & Gynecological thermography	NO	Skin temperature thermal imaging was proposed as an improvement to conventional body (oral) temperature methods and as a means to portray the temperature of the entire body surface. Conditions examined include breast, placental localisation and pregnancy with the objective of identifying thermographic ‘hot spots’ commensurate with pathological lesions. No measures of the relationship between hot spots on thermography and concomitant changes in cutaneous blood flow were made.
Simoes et al., 2012 [61]	Thermal skin reference values in healthy late pregnancy	NO	Healthy pregnant women during the third trimester. Postpartum women were not included. Objective: assessment of distribution pattern of thermal symmetry using infrared thermography with regions of interest over the abdomen. By contrast to current literature in healthy adults, temperature of lower abdominal ROIs were lowest in healthy pregnant women of normal weight and highest in those in obese categories. No explanation is provided. No measures of cutaneous blood flow were undertaken.

## Appendix A

**Table A1:** Boolean operators under four facets “maternal OR postpartum”, “abdominal skin”, “blood flow or perforators” and “thermal imaging”.

#Set	Query	Limiters/Expanders
S1	maternal OR mother * OR postnatal OR postpartum OR after childbirth	Limiters—human; Expanders—apply equivalent subjects; Search modes—Boolean/phrase.
S2	abdominal OR skin OR cutaneous OR subcutaneous	
S3	blood flow OR perforator* OR skin perforation	
S4	(S1 AND S2 AND S3) NOT Fet* NOT newborn NOT infant NOT placenta	
S5	abdom* AND (thermal imaging OR thermograph*) NOT fet* NOT newborn NOT infant NOT placenta NOT mast* NOT breast	
S7	S5 OR S6	

* wildcard symbol to broaden search

## Appendix B. Charting of Key Review Items

**Table A2:** Charting Form.

1.	Author
2.	Year of publication
3.	Title
4.	Publication: Journal/Book
5.	Source origin/country
6.	Aims/Objectives/Purpose
7.	Study population
8.	Sample size
9.	CONCEPT: Phenomenon of interest relating to the measurement of abdominal temperature and cutaneous blood perfusion
10.	Additional references found from citation list
11.	Methods used
12.	Equipment and cameras
13.	Calibration
14.	Camera resolution
15.	Duration of use
16.	Any comparator?
17.	CONTEXT: medical specialty, geography, clinical, experimental
18.	Whether meets aim of review—caesarean section Yes/No
19.	Outcomes and details of the results (e.g., how measured)
20.	Explanation
21.	Quantitative mapping YES NO
22.	Qualitative mapping YES NO
23.	Temperature values (°C) if any. How documented?
24.	Key findings that relate to scoping review questions
25.	Actions
